# Insights into anti-tumor immunity *via* the polyomavirus shared across human Merkel cell carcinomas

**DOI:** 10.3389/fimmu.2023.1172913

**Published:** 2023-05-23

**Authors:** Saumya Jani, Candice D. Church, Paul Nghiem

**Affiliations:** ^1^ Department of Medicine, University of Washington, Seattle, WA, United States; ^2^ Fred Hutchinson Cancer Center, Seattle, WA, United States

**Keywords:** Merkel cell carcinoma, Merkel cell polyomavirus, skin cancer, anti-tumor T cells, immunotherapy, oncoproteins

## Abstract

Understanding and augmenting cancer-specific immunity is impeded by the fact that most tumors are driven by patient-specific mutations that encode unique antigenic epitopes. The shared antigens in virus-driven tumors can help overcome this limitation. Merkel cell carcinoma (MCC) is a particularly interesting tumor immunity model because (1) 80% of cases are driven by Merkel cell polyomavirus (MCPyV) oncoproteins that must be continually expressed for tumor survival; (2) MCPyV oncoproteins are only ~400 amino acids in length and are essentially invariant between tumors; (3) MCPyV-specific T cell responses are robust and strongly linked to patient outcomes; (4) anti-MCPyV antibodies reliably increase with MCC recurrence, forming the basis of a standard clinical surveillance test; and (5) MCC has one of the highest response rates to PD-1 pathway blockade among all solid cancers. Leveraging these well-defined viral oncoproteins, a set of tools that includes over 20 peptide-MHC class I tetramers has been developed to facilitate the study of anti-tumor immunity across MCC patients. Additionally, the highly immunogenic nature of MCPyV oncoproteins forces MCC tumors to develop robust immune evasion mechanisms to survive. Indeed, several immune evasion mechanisms are active in MCC, including transcriptional downregulation of MHC expression by tumor cells and upregulation of inhibitory molecules including PD-L1 and immunosuppressive cytokines. About half of patients with advanced MCC do not persistently benefit from PD-1 pathway blockade. Herein, we (1) summarize the lessons learned from studying the anti-tumor T cell response to virus-positive MCC; (2) review immune evasion mechanisms in MCC; (3) review mechanisms of resistance to immune-based therapies in MCC and other cancers; and (4) discuss how recently developed tools can be used to address open questions in cancer immunotherapy. We believe detailed investigation of this model cancer will provide insight into tumor immunity that will likely also be applicable to more common cancers without shared tumor antigens.

## 1 Introduction

It is well established that the ability of T cells to target tumor cells is central to controlling and eliminating cancer (1). In contrast, details of the relative roles of cancer-specific lymphocytes and innate immune cells in the short- and long-term control of cancer is highly complex and not fully understood. Cancer immunotherapy has dramatically transitioned in the past decade from an understudied, empiric field to a dominant and mainstream scientific discipline. Nevertheless, enormous challenges remain. A major roadblock to improved understanding of cancer immunology is an inability to readily identify cancer-specific T cells due to the “private” (patient specific) nature of most tumor antigens. Personalized mutations and unique neoantigens expressed by most tumors require custom reagents for each tumor to study cancer-specific T cells between patients, making this process tedious and not readily amenable to studying a larger cohort of patients.

We propose that Merkel cell carcinoma (MCC) is a model cancer in which to study T cell and B cell responses because of the shared, highly immunogenic oncoproteins that drive this cancer. MCC is a rare but aggressive cutaneous neuroendocrine carcinoma with an incidence of about 3,200 new cases per year in the US (2). Initial observations that HIV+ patients had a >10-fold increased risk of MCC, suggested that this was an immune sensitive cancer and might be driven by a pathogen (3–5). In 2008, the Pittsburgh-based laboratory of Patrick Moore and Yuan Chang made the landmark discovery of a novel human DNA polyomavirus that was clonally integrated into a chromosome of the host (tumor) cell (6).

Merkel cell polyomavirus (MCPyV) is one of about a dozen human polyomaviruses, all of which show evidence of largely asymptomatic infection of more than half of healthy persons by adulthood (7). Accordingly, the majority of MCC cases (70-80%) are driven by MCPyV oncoproteins (8, 9). Of note, MCPyV is highly conserved across individuals, with very minimal amino acid polymorphisms. This also holds true across MCC tumors, meaning there is minimal antigenic variability between patient tumors (10, 11). The remaining ~20% of MCCs are caused by extensive ultraviolet (UV)-induced mutations (median of 1121 protein-coding somatic single nucleotide variants per exome) resulting in numerous immunogenic neoantigens expressed by tumor cells (12–14). Strikingly, both MCPyV-driven and UV-driven MCCs have a high (~50%) response rate to anti-PD-(L)1 therapies, suggesting that both etiologies are immunogenic in nature (15–17). The fact that two very distinct processes (UV-mutational and virus-induced) can lead to a histologically identical, aggressive, fast growing, immune-sensitive tumor provides numerous opportunities for insight into cancer immunobiology.

Several lines of evidence have demonstrated that expression of small and large T antigens of MCPyV is necessary in an ongoing way for virus-driven MCC growth and survival, essentially rendering the tumor cells “addicted” to these oncoproteins, which can therefore not be lost (18). This unique situation provides several advantages relevant to cancer immunobiology. Like any tumor antigen, viral oncoproteins are processed into peptides that are then presented by major histocompatibility complexes (MHCs) on the surface of tumor cells. These MHC-peptide complexes are in turn recognized by cancer-specific T cells. The fact that MCPyV oncoproteins are shared between patients allows for development of a suite of reagents that can identify tumor-specific immune responses among an entire cohort. Because the immune response can be studied across multiple patients, data gathered in virus-driven MCC can be linked to the patients’ clinical outcomes and the relative significance of various immune-associated characteristics of interest can be determined.

Herein, we describe insights gleaned from virus-driven MCC by comparing the tumor microenvironments (TME) and T cell characteristics of patients who experience different outcomes. We will also review immune evasion and immunotherapy resistance mechanisms in MCC and solid tumors more broadly, as well as explore new therapeutic opportunities. Finally, we will discuss recent and anticipated technologies that help address open questions in cancer immunology.

## 2 Study of virus-driven MCC overcomes typical limitations imposed by private tumor antigens

Most cancers are driven by patient-specific mutations, and the resulting neoantigens are unique to each patient and vary in immunogenicity. While neoantigens can be rapidly identified by current whole exome sequencing (WES) and bioinformatic tools, extending this to create and validate tools (peptide-MHC tetramers, described below) that identify T cells that can recognize these neoantigens remains cumbersome and logistically infeasible. This is relevant as most predicted peptides are not immunogenic and do not identify cancer-specific T cells, requiring screening of large amounts of epitopes to identify T cells (19, 20). Additionally, the unpredictable and varied levels of immunogenicity prevent comparisons of cancer-specific T cells across patients. To avoid the complications related to personalized tetramer-based identification of cancer-specific T cells, approaches involving response to *in vitro* stimulation and expression of activation markers have been developed (21, 22). While identification of cells with such activation markers is relatively easy, they are not limited to tumor antigens, resulting in capture of activated bystander T cells within the target population. Together, the inability to reliably identify tumor neoantigen-specific T cells between patients prevents the use of high-throughput or bulk approaches that would be feasible for clinical use.

**Figure 1:**
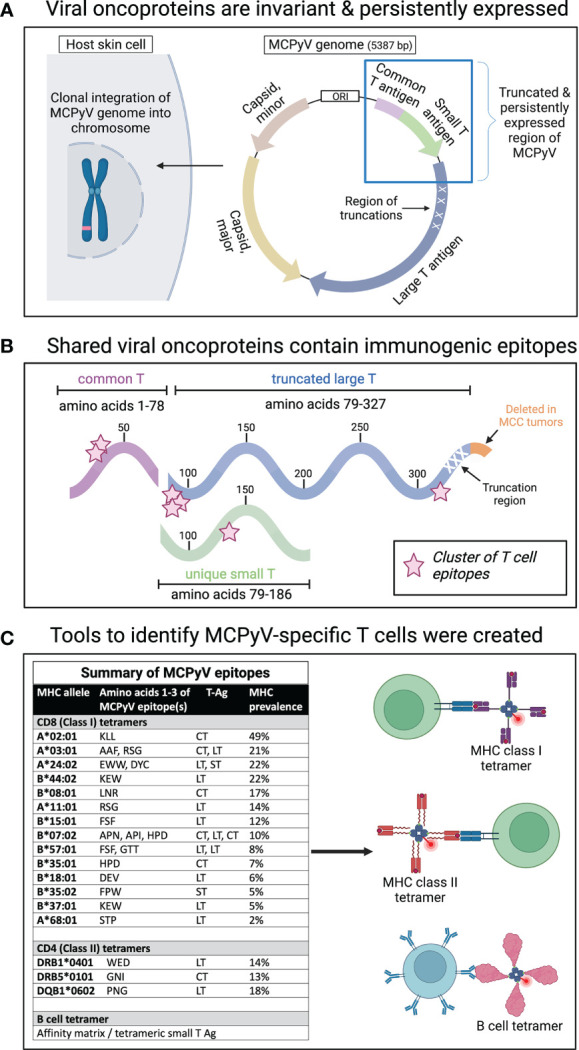
Virus-driven MCC is a unique model cancer for studying anti-tumor T cell responses. **(A)** Virus-driven MCC arises when MCPyV undergoes a truncation and integrates into the host genome. The integration site is thought to be random as it varies greatly among patients. Following the truncation and integration, two oncoproteins (large and small T antigens), which share a common region, are persistently expressed and drive tumorigenesis. **(B)** These viral oncoproteins are small (~400 amino acids), and this antigenic space has been rigorously studied to identify immunogenic peptides presented by common MHC types. The areas of immunogenicity are highlighted with pink stars. **(C)** Based on these functional studies, multimer tools to identify cancer-specific T and B cells have been developed and validated. The immunogenic peptides, the MHC molecules that can present them, the relevant oncoprotein region, and an approximation of the prevalence of these MHC molecules in patients with MCC are summarized in the table.

MCC, on the other hand, is driven by MCPyV in 80% of cases and has an exceptionally low tumor mutational burden (TMB) (23). This means that MCPyV oncoproteins are largely responsible for driving tumorigenesis and most cancer-specific T cells recognize MCPyV oncoproteins. In addition, MCPyV oncoproteins are small (approximately 400 amino acids in length) and invariant between patients (**Figure 1A**). Careful annotation of the integrated sequence has allowed for functional studies that identified MHC-restricted epitopes (**Figure 1B**) **(** 21). Initial characterization of MCPyV immunogenicity was accomplished by stimulating blood and tumor samples from MCC patients with T antigen peptides. These studies revealed T cell responses to shared antigens across MCC patients. Based on these data, tetramer reagents were created. Tetramers consist of four MHC molecules containing a previously identified MCPyV peptide conjugated to a tetrameric streptavidin-biotin scaffold (**Figure 1C**). These molecules bind to MCPyV-specific T cells in a highly avid and sensitive manner and allow study of T cell responses across MCC patients in an efficient and reproducible way.

Tetramers offer additional advantages. By relying on T cell receptor (TCR) specificity, instead of functional readout to identify cancer-specific T cells, tetramers allow recognition of T cells that have lost cytotoxic capabilities due to chronic antigen stimulation. Indeed, in patients with a persistent or heavy disease burden, T cells are often identifiable with tetramers, but not through stimulation assays that measure interferon-gamma (IFN-γ) or interleukin-2 (IL-2) secretion as a means of detecting the presence of cancer-specific T cells (24). This is because cancer-specific T cells are often dysfunctional and incapable of expressing the activation markers or secreted effector cytokines used to detect them in such activation-based assays (24). Furthermore, they can be combined with other technologies to more deeply characterize cancer-specific T cells. Adding fluorophore-conjugated tetramers to flow cytometry panels has shown that tetramer-positive cells express higher levels of immune checkpoint markers such as programmed cell death protein 1 (PD-1) and T cell immunoglobulin and mucin domain (TIM-3), providing further evidence of dysfunction (25). Adding DNA barcode-conjugated tetramers facilitates identification of T cell specificity and links this data to single-cell sequencing-based approaches. This can allow us to characterize the epigenetic and transcriptional state of tumor-specific T cells (26–28). The next section summarizes what we have learned about the importance of cancer-specific T cells in MCC using these tools.

## 3 Lessons learned from tumor-specific lymphocytes in MCC

Given that the risk of MCC is often more than 10-fold higher in immune suppressed patients (while it is only ~2-fold higher than patients with malignant melanoma), the role of the immune system in responding to this cancer was of particular interest (29). In 2010, brisk infiltration of CD8+ T cells into MCC tumors was found to be predictive of improved MCC-specific survival, helping to explain the importance of tumor-specific immune responses for this cancer (30). However, it was not known whether the infiltrating T cells were capable of recognizing MCPyV oncoproteins. Once the viral sequence for MCPyV was described, investigation of patient blood and tumors demonstrated that T cells specific for the oncoproteins were present in MCC patients, but absent in corresponding specimens from healthy controls (31, 32). Furthermore, it has been observed that when tumors are effectively treated (surgically removed or fully respond to treatment) and the source of tumor antigens is no longer present, the number of MCPyV-specific T cells quickly drops (25). Considering that MCPyV-driven MCC has a low TMB (~12.5 mutations per exome versus 1,121 for UV-driven MCC), these data suggest that the relevant tumor antigens for the immune system to effectively target are indeed the viral antigens (12).

It is well established that chronic exposure to an antigen, and consequent activating signals, can cause T cells to become dysfunctional (33). Dysfunctional T cells can be identified by their expression of activation and exhaustion markers, including co-expression of PD-1, TIM-3 and others. When PD-1 on a T cell is engaged by its ligand, it significantly restrains the function of that cell. Consistent with this being a dominant immune evasion mechanism in MCC, oncoprotein-specific T cells in MCC patients often express PD-1. In over half of MCC tumors, programmed death-ligand 1 (PD-L1) is expressed by tumor cells and/or antigen presenting cells (25, 34). These data suggested that MCC tumors should be amenable to PD-1 pathway blockade therapies and provided a strong rationale for clinical trials to test this approach. Indeed, clinical trials of anti-PD-(L)1 as first-line treatment in MCC patients resulted in very high objective response rates of 56–62% (16, 17, 35). While many of these responses were durable, in the end, fewer than half of MCCs patients have persistent benefit from PD-1 pathway blockade. Because of the highly immunogenic nature of this cancer and its shared tumor antigens, this is an excellent opportunity to characterize the basis of primary and acquired resistance to immunotherapy.

### 3.1 Characteristics of tumor-infiltrating MCPyV oncoprotein-specific CD8 T cells linked to improved outcomes

**Figure 2:**
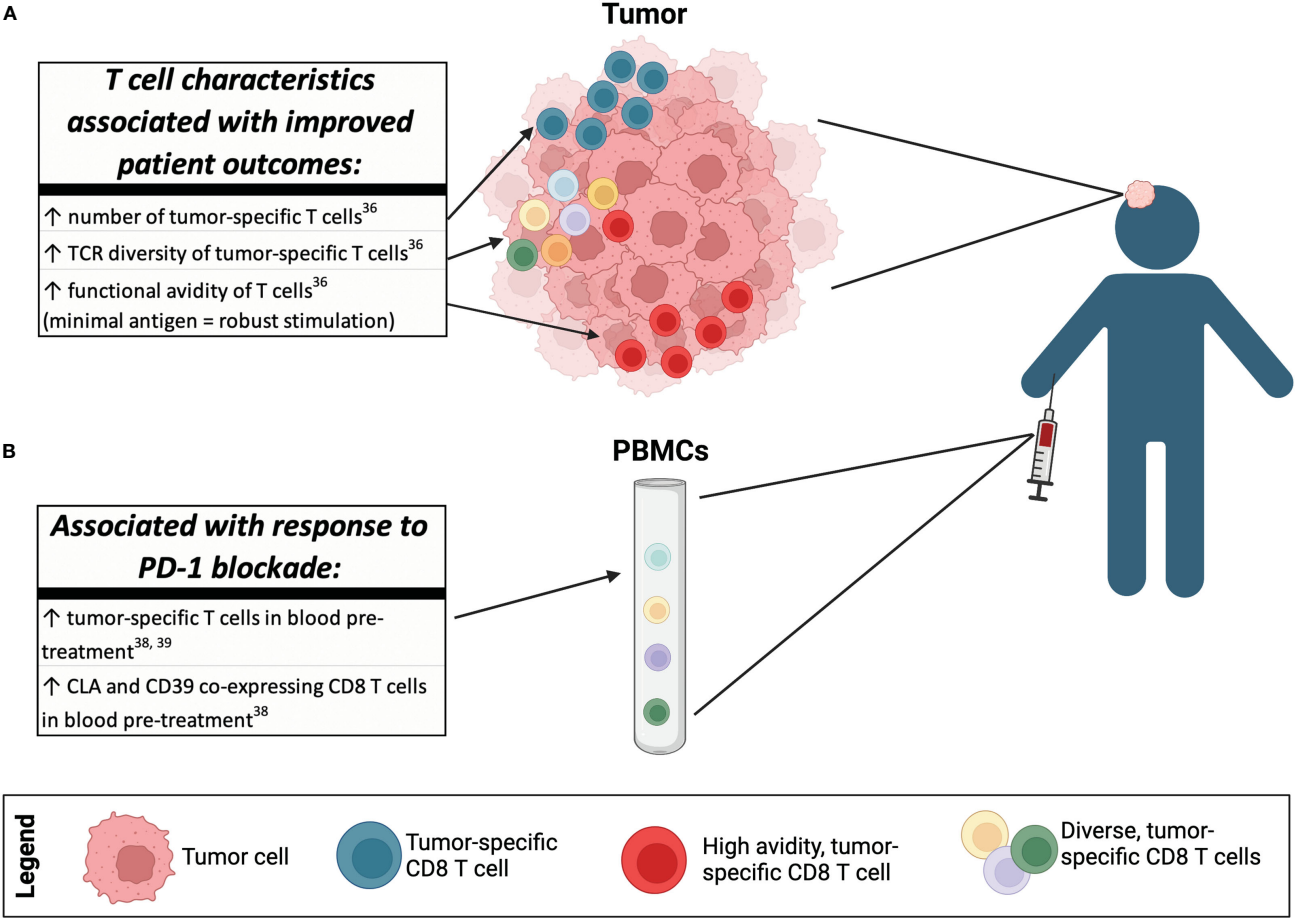
Insights into anti-tumor T cell responses gained by studying virus-driven MCC. Several lines of evidence have shown that virus-driven MCC is targetable by CD8 T cells and the presence of the MCPyV oncoprotein-specific T cells in patients can be linked to improved clinical outcomes. **(A)** Increased number of MCPyV oncoprotein-specific T cells, diverse T cell clonotypes that target MCPyV oncoprotein antigens, and CD8 T cells with high functional avidity found in the tumor are linked to improved patient outcomes in the absence of immunotherapy. **(B)** Circulating peripheral blood T cell characteristics that are associated with response to immunotherapy include the presence of MCPyV oncoprotein-specific CD8 T cells before initiation of anti-PD-1 therapy as assessed by MHC-peptide tetramer, and the presence of CD8 T cells that co-express CD39 and CLA before initiation of anti-PD-1 therapy.

To determine the link between MCPyV oncoprotein-specific CD8 T cells and clinical outcomes, T cells from patient tumors and blood were characterized, and differences between patients with divergent outcomes were assessed (36). Intratumoral T cell clones specific for a single MCPyV oncoprotein epitope (KLLEIAPNC) were tracked in a patient cohort by their unique complementarity determining region 3 sequences of the TCRβ chain (**Figure 2A**) **(** 36). Frequency of T cell clones specific for this KLL epitope found within tumors was significantly greater in patients with extended survival compared to those with fewer clonotypes (36). Patients with >5 unique clonotypes had greater survival (p = 0.0051) and a higher chance of recurrence-free survival after definitive treatment than patients without diverse cancer-specific T cells (36). Additionally, patients who did not experience recurrences had more avid T cells (T cell clones that could respond well to low concentrations of peptide in a functional IFN-γ assay) compared to patients who experienced recurrence (36). Taken together, these data suggest that the presence of diverse, prevalent, and highly functional T cells that target tumor antigens are linked to improved patient outcomes. One approach to directly test the efficacy of these cancer-specific cells is being explored in a clinical trial of autologous, TCR-transgenic T cells that employ a highly avid A02-KLL-specific TCR (NCT03747484).

### 3.2 Links between peripheral blood circulating T cells and immune checkpoint inhibitor responses

A predictive biomarker for responsiveness to immunotherapy is needed to help determine the best therapeutic agents for a patient to experience long term control of their cancer. While expression of PD-L1 in the tumor, microsatellite instability (MSI) and TMB are used as predictive biomarkers of response to anti-PD-1 agents for various cancers, none have been shown to be clinically relevant for MCC (37). In a study of advanced MCC patients who received first-line pembrolizumab (NCT02267603), oncoprotein-specific CD8 T cells tracked with responsiveness wherein responders (n = 13) had more abundant cancer-specific T cells in blood at baseline compared to non-responders (n = 4) (38). Analogously, it was also reported that the presence of baseline circulating oncoprotein-specific CD8 T cells significantly correlated with progression-free survival in patients receiving neoadjuvant anti-PD-1 (NCT02488759) (**Figure 2B**) **(** 39). Although both of these studies had a small number of available specimens and additional cohort(s) are needed to confirm these findings, both suggest improved outcomes for MCC patients who have more circulating oncoprotein-specific T cells at baseline. While it has long been appreciated that anti-tumor CD8 T cells are critical for control of cancer, these two studies are the first to show that MCC patients who have existing MCPyV oncoprotein-specific T cells at the start of therapy were more likely to respond. For patients who don’t have circulating MCC-specific T cells prior to initiation of anti-PD-(L)1 therapies, adoptive cell therapies and therapeutic vaccines could be administered to increase the chances of a favorable response.

## 4 MCC requires multiple immune escape mechanisms at play

As discussed in the introduction, there are numerous lines of evidence that suggest that MCC is an immunogenic cancer, regardless of whether it was induced by MCPyV (viral epitopes) or UV exposure (neoantigens). This immunogenicity suggests that the immune system will detect the tumor at an early stage of development and deploy effective anti-tumor strategies, if robust immune evasion mechanisms are not employed. Indeed, individuals with a compromised or suppressed immune system are at a 10-50- fold greater risk of developing MCC, depending on the types and severity of immunosuppression (5, 40, 41). However, at least 90% of MCC cases arise in immune competent persons, suggesting that a vast majority of these tumors do have the capacity to evade an intact immune system. Indeed, MCC tumors have been observed to use one or more of these immune evasion approaches detailed below: (1) induce an immunosuppressive TME; (2) prevent T cell infiltration into the tumor; (3) inhibit tumor engagement with T cells; and (4) induce T cell dysfunction.

### 4.1 Immunosuppressive tumor microenvironment

Mechanisms by which MCC tumors induce an immunosuppressive microenvironment include suppression of innate immune danger signals and the presence of immune suppressive cells. Toll-like receptor 9 (TLR9) is an intracellular toll-like receptor that activates an inflammatory immune response upon recognizing foreign DNA, a potent danger signal to indicate a cell has been infected by a virus (42). MCPyV T antigens inhibit CCAAT/enhancer binding protein (C/EBP) transcription factor, which leads to downregulation of TLR9 (43). Indeed, a clinical study of cystic fibrosis patients demonstrated lower levels of TLR9 in patients harboring MCPyV (44). Thus, downregulation of TLR9 in MCC tumors could prevent recruitment of immune cells and may contribute to tumor survival. Additionally, the TME can induce immunosuppressive phenotypes in infiltrating immune cells, including macrophages and T regulatory cells.

While complex and controversial, macrophages can be grouped into two broad categories, M1 and M2. M1 macrophages are classically activated by IFN-γ or lipopolysaccharide (LPS) and secrete proinflammatory cytokines to protect against pathogens. M2 macrophages, on the other hand, are activated by exposure to other cytokines, including interleukin-4 (IL-4), interleukin-10 (IL-10), and transforming growth factor beta 1 (TGF-β1), and secrete immunosuppressive cytokines and factors that promote angiogenesis and tissue repair (45, 46). M2 macrophages are frequently found in MCC tumors and support tumor growth (46, 47). Furthermore, tumor cells and innate immune cells in the TME also secrete chemokines that recruit T regulatory cells (CCL17/22, CCL5, CCL28 and CXCL9/10/11), expand T regulatory cells (TGF-β, IL-10), and convert conventional T cells into T regulatory cells (TGF-β and adenosine) (48). These cells typically inactivate CD8 T cells and antigen-presenting cells to suppress an overactive immune response and prevent immune-mediated damage. While T regulatory cells are enriched in MCC tumors, their role in tumorigenesis and patient outcome is unclear, as there is no striking association between their presence and altered outcomes (47, 49).

### 4.2 Lack of T cell infiltration

**Figure 3:**
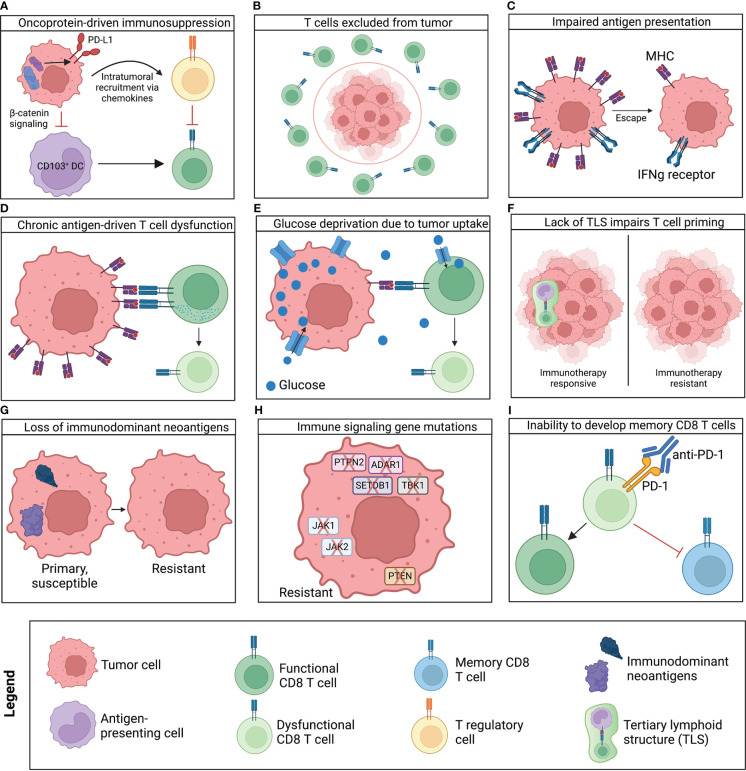
Summary of mechanisms of resistance to immunotherapy identified to-date. Several mechanisms can prevent response to immunotherapy altogether (primary resistance), while other mechanisms are acquired later in the disease course and lead to tumor relapse (secondary resistance). **(A)** Oncoproteins can induce immune suppressive environments that recruit tumor-promoting macrophages and T regulatory cells, prevent infiltration of antigen-presenting cells, and prevent infiltration and priming of effector T cells. **(B)** Tumor cells can prevent T cell recruitment *via* downregulation of inflammatory pathways and cell surface integrins (e.g., E-selectin) that mediate T cell entry into inflamed tissues. **(C)** Tumors often downregulate or develop mutations in genes responsible for antigen presentation (e.g., MHC, IFN-γ receptor), preventing tumor engagement with T cells. **(D)** Chronic exposure to their cognate antigens leads to activation of evolutionarily protective, immunosuppressive mechanisms that convert effector T cells to a hypofunctional phenotype. **(E)** Increased glucose uptake by the tumor leads to T cell activation in a nutrient-poor environment, which seems to prime T cells to attain a hyporesponsive phenotype. This phenotype cannot be reversed even if T cells are subsequently stimulated in nutrient-rich conditions. **(F)** Lack of TLS can prevent appropriate T cell priming and is linked to poor outcomes and poor response to immunotherapy. **(G)** Cancer and immune cells co-exist in balance with each other. Thus, immune pressure can lead to deletion of immunogenic neoantigens not required for tumor survival. This “hides” the tumor from the immune system. **(H)** Tumors can also downregulate or mutate cell surface receptors and intracellular proteins responsible for recognizing and responding to immune effector signals (e.g., PTPN2, ADAR1, SETDB1, TBK1, JAK1/2, and PTEN), thus preventing immune-mediated cancer cell death. **(I)** ICI has been demonstrated to re-invigorate the effector functions of T cells but may not induce memory cell formation. This would allow metastases and microtumors to grow after initial disease has been controlled.

In addition to creating an immunosuppressive environment, tumors can also employ strategies that prevent T cell infiltration (**Figure 3B**), such as downregulation of stimulator of interferon genes (STING) and E-selectin. MCC tumors silence STING, a molecule that senses DNA damage, activates a cytokine response, and recruits cancer-specific T cells to the TME. When STING expression was artificially restored in a human MCC cell line that was then co-cultured with MCPyV-specific T cells, the cancer-specific T cells demonstrated increased cytokine production, migration, and tumor cell killing. This implies that downregulating STING allows the tumor to prevent a significant innate immune mechanism of T cell recruitment (50). In an actual human tumor, injection of a small molecule agonist of STING signaling induced infiltration of MCPyV-specific T cells and regression of multiple injected and non-injected tumors (51). MCC tumors have also been shown to downregulate E-selectin, an integrin critical for T cell homing to the skin, *via* local nitric oxide production. Tumors with increased amounts of nitrotyrosine, a stable biomarker of nitric oxide-induced reactive nitrogen species, had lower E-selectin expression, and consequently, lower T cell infiltration (52). The expression of the ligand for E-selectin, cutaneous lymphocyte antigen (CLA), needs to be further elucidated. Some studies found that a higher percentage of MCPyV-specific T cells express CLA when compared to T cells that target viruses that do not infect the skin (38, 52). A different study found low expression of CLA in cancer-specific T cells and corresponding reduced T cell infiltration into the TME of these patients (49).

### 4.3 Inhibition of immune cell engagement with the tumor

If efforts to prevent T cells from entering the TME are unsuccessful, MCC tumors can downregulate cell surface receptors that engage cytotoxic immune cells (**Figure 3C**). Cytotoxic CD8 T cells detect infected or cancerous cells by their TCRs binding to peptides presented on MHC complexes on the cells of interest or professional antigen-presenting cells. Typically, these peptide-MHC complexes are translocated to the cell surface after intracellular proteins are degraded, bound to MHC class I proteins (53). MCPyV oncoproteins interfere with this process by downregulating immunoproteasome genes (LMP2 and LMP7), transporters associated with antigen processing (TAP1 and TAP2), and antigen presentation molecules (MHC class I and β2 microglobulin) (54). The vast majority of MCC tumors (85%) have partial or complete downregulation of class I MHC on their surface relative to surrounding stromal structures (55). Specifically, MHC class I molecules are transcriptionally downregulated by L-Myc, the levels of which are markedly upregulated by the MCPyV oncoproteins (56). The small T antigen binds to L-Myc and recruits it to the EP400 histone acetyltransferase and chromatin remodeling complex to block expression of class I MHC (56). Class I MHC downregulation also confers transcription level resistance to adoptive T cell therapies reliant on presentation of peptide-MHC on MCC tumor cells for anti-tumor responses (57). Correspondingly, this class I MHC downregulation can be pharmacologically reversed by histone deacetylase inhibitors and interferons (54, 55, 58).

In addition to T cells, natural killer (NK) cells also possess cytotoxic potential. This is of particular importance because of the high frequency of class I MHC downregulation in MCC and the fact that NK cells are typically activated by lack of class I MHC on the surface of target cells. NK cells engage target cells based on a balance of activating and inhibitory cell surface receptors and costimulatory molecules (CD40, CD80, and CD86) (59, 60). MCC tumors epigenetically inhibit transcription and translation of two key natural killer group D ligands, MICA and MICB (61). Expression of these ligands on a cell’s surface normally sends a “kill me” signal to NK cells. MICA and MICB can also be upregulated by histone deacetylase inhibitors, which have been explored as therapeutic targets in MCC due to their ability to upregulate both “kill me” signals and class I MHC (61). Indeed, in a small clinical trial of NK cells +/- IL-15, 2 of 6 MCC patients with immunotherapy refractory disease experienced clinical benefit and had indications of increased inflammation in their tumors (62). These data suggest that MCC can be sensitive to NK cell killing and therapeutics that recruit NKs cells into the TME should be further explored.

### 4.4 Induction of T cell dysfunction

To prevent tissue damage based on an immune response that persists unabated, immune cells express inhibitory receptors during activation, allowing inflammation to be attenuated after the acute response (**Figure 3D**). Chronic signaling through the TCR leads to elevated expression of these inhibitory receptors and facilitates irreversible differentiation to a hyporesponsive state *via* epigenetic alterations. This dysfunctional phenotype interferes with both mounting of an effective inflammatory response and developing stem-like and memory T cells. Indeed, MCC-specific T cells express greater levels of inhibitory receptors (PD-1, cytotoxic T lymphocyte-associated protein-4 (CTLA-4), TIM3, lymphocyte activation gene-3 (LAG-3), and T cell immunoreceptor with Ig and ITIM domains (TIGIT)) and lower levels of activation markers (CD25 and CD69) than bystander T cells (49). Bystander T cells are defined as either T cells present in healthy skin or T cells specific to other common viruses. MCC tumors take advantage of this mechanism by expressing immune checkpoint molecules, specifically PD-L1 and PD-L2, that can inactivate T cells that recognize the tumor (34, 49, 63). Along the same lines, several groups have discovered that a specific subset of PD-1+ cancer-specific T cells (expressing stem cell markers, TCF1 and CXCR5) do expand in response to anti-PD-1 therapy and are largely responsible for controlling the tumor (64–70). It is postulated that these progenitor exhausted cancer-specific T cells recognize antigens that may be expressed at lower levels or do not bind class I MHC as avidly, allowing these T cells to receive fewer activation signals and avoid a more irreversible dysfunctional phenotype (69, 71). It would be pertinent to determine whether this subset of cells could serve as a biomarker of response to immunotherapy in MCC.

## 5 A major open question: Why do some patients not benefit from immunotherapy?

Although MCC-specific T cells typically express inhibitory receptors targeted by immunotherapy (i.e., PD-1, CTLA-4, TIM-3, LAG-3), only about half of patients persistently respond to immune checkpoint inhibition (ICI). To address immunotherapy-refractory disease, it is critical to understand the mechanisms underlying response or resistance to immunotherapy. In addition to the previously reported MCC-specific immune evasion mechanisms described above, several resistance mechanisms have been demonstrated in other cancers and may also underlie immunotherapy resistance in MCC. Below is a summary of such mechanisms documented in other cancers to underlie primary resistance (no response at all) and secondary resistance (relapse of tumor following a response) (**Figure 3**).

### 5.1 Primary resistance

Many of the identified mechanisms of primary resistance involve anti-tumor T cells: (1) lack of cancer-specific T cells in the right place; (2) inability of existing T cells to mount anti-tumor immune responses; (3) inability to generate new T cell responses; and (4) tumor resistance to effector cytokines.

#### 5.1.1 Lack of cancer-specific T cells in the right place

Given that a key target of PD-(L)1 blockade is cancer-specific T cells, it is imperative that such T cells are in fact present in the patient. Indeed, a randomized controlled clinical trial studied the efficacy of neoadjuvant vs. adjuvant treatment in 313 patients with resectable stage III-IV melanoma. Event-free survival was significantly higher in the neoadjuvant group 2 years after treatment (72). Both intratumoral T cells and the source of relevant cancer antigens (tumor cells) are removed during surgical resection and are thus absent when ICI is given in the adjuvant setting. The simplest explanation is that the presence of tumor at the time of immunotherapy initiation elicits a more robust immune response than if the tumor had already been removed. In contrast, other studies have found that chronic antigen stimulation by the tumor drives intratumoral T cells to dysfunction and that peripheral T cells may play a larger role in responding to anti-PD-(L)1 treatment (73, 74).

In a study of 11 patients with basal cell carcinoma, sequential biopsies of a patient’s tumor before and after immunotherapy were analyzed, revealing that expanded T cell clones in post-therapy samples were not present in the pre-treatment sample (73). This suggests that the T cells that respond to ICI may have recently infiltrated the tumor. In addition, recent studies demonstrated that the degree of expansion of cancer-specific CD8 T cells in peripheral blood predicts response to PD-L1 blockade in patients with metastatic urothelial carcinoma (74). Similarly, the frequency of activated (PD-1+, KLRG1-) peripheral CD8 T cells predicts pathologic response in oral cancer (75). This implies that peripheral blood could serve as a reservoir of cancer-specific T cells that infiltrate the tumor after immunotherapy. It is likely a combination of intratumoral and peripheral cancer-specific T cells that respond to ICI, a balance that likely differs between patients.

Finally, it is possible that some patients do not have functional peripheral or intratumoral cancer-specific T cells. It has been observed that patients who do not respond to immunotherapy have more turnover of intratumoral T cells, implying that their T cells are not recognizing the cancer and receiving signals to stay/proliferate and fight (76). Additionally, this lack of T cells may be a consequence of minimally immunogenic neoantigens. Indeed, cancers that have a high TMB, either because of non-synonymous mutations or MSI as a result of inability to repair DNA mismatches, are more responsive to immunotherapy (77–81).

#### 5.1.2 T cell generation and priming

If cancer-specific T cells are not present or are terminally exhausted, new T cells recruited from a naïve population could be primed and expanded to exert anti-tumor immunity. Given that thymic function gradually decreases with age and new T cells are rarely produced in older persons, this may contribute to a lack of T cells capable of responding to PD-(L)1 blockade (82). Additionally, existing naïve cancer-specific T cells may not be primed and activated appropriately due to TME or tumor draining lymph node conditions (**Figure 3F**). For example, it has been observed that β-catenin signaling decreases the recruitment of antigen-presenting CD103+ dendritic cells, which in turn reduces priming and activation of naïve cancer-specific T cells that enter the TME (83). Additionally, several studies have shown that formation of tertiary lymphoid structures (TLS) within the tumor correlates to improved response to immunotherapies (84–86). It is possible that TLS are where primary or secondary immune responses are generated and thus play a role in the priming and clonal expansion of cytotoxic CD8 T cells (87, 88). Thus, deficiencies of cytokines that promote formation of effective TLS could contribute to resistance to cancer immunotherapy. Both antibody and T cell frequencies track with MCC tumor burden (25, 89) suggesting a coordinated immune response and that the presence of TLS in MCC could be predictive of response.

#### 5.1.3 Tumor resistance to effector cytokines

Four genes have been implicated in aiding immune evasion or conferring resistance to anti-PD-1 treatment: protein tyrosine phosphatase non-receptor 2 (PTPN2), RNA editing enzyme ADAR1 (Adenosine Deaminase Acting on RNA 1), H3K9 methyltransferase SETDB1 (SET Domain Bifurcated Histone Lysine Methyltransferase 1), and tank-binding kinase 1 (TBK1) (**Figure 3H**) **(** 90–93). The PTPN2 phosphatase is involved in multiple signaling pathways, including negative regulation of IFN-γ signaling (90). Loss of PTPN2 increases IFN-γ mediated antigen presentation and growth suppression in melanoma and colon carcinoma murine models (90). Additionally, tumors lacking PTPN2 contain CD8 T cells with increased expression of granzyme B, a marker of activated, cytotoxic T cells (90). ADAR1 is an adenosine deaminase that prevents the sensing of endogenous double-stranded RNA (91). Thus, loss of ADAR1 increases cellular sensing of double-stranded RNA, and consequently inhibits tumor growth and increases inflammation (91). ADAR1 loss sensitizes tumors to IFN-γ signaling and overcomes resistance to anti-PD-1 immunotherapy (91). SETDB1, a histone methyltransferase, represses domains within the open gene compartment that contain transposable elements and immune genes (92). Loss of SETDB1 increases the activity of transposable element-mediated regulatory and immune stimulatory genes, in addition to increasing transposable element-specific cytotoxic T cell responses (92). TBK1 kinase coordinates the innate immune response to viruses by integrating signals from pattern recognition receptors and cytosolic nucleic acid sensors, and regulating activation of type I interferons and interferon-stimulated genes (93). Using PDX and organoid models, the authors demonstrated that loss of TBK1 lowered the threshold of immune cell-secreted effector cytokines needed to kill tumor cells and sensitized tumors to anti-PD-1 therapy (93). While these genes have not been studied in the context of MCC, these may be effective immunotherapy targets or might synergize with existing immunotherapies. Additional studies of MCC patient tumor biopsies are needed.

#### 5.1.4 Emerging mechanisms: Physical removal of anti-PD-1 antibodies, gut microbiome, and cytotoxic CD4+ T cells

In addition to the CD8 T cell-based mechanisms described above, additional insights are emerging from analyses of specimens collected from clinical trials and mouse models. Three promising findings regarding primary immune resistance are summarized here.

In mouse models derived from colon carcinoma, melanoma, or lung adenocarcinoma cell lines, *in vivo* imaging revealed that PD-1- macrophages stripped anti-PD-1 antibodies from the surface of CD8+ T cells (94). This removal was dependent on the Fcy receptors present on the macrophages and Fc region of the antibodies, suggesting that engineering the Fc region of anti-PD-1 treatments could increase the time of antibody engagement with cancer-specific CD8 T cells (94).

The gut microbiome is known to influence immune responses, and recent studies have shown that the microbiome also influences response to immunotherapy (95–97). Indeed, microbiome profiling has revealed that gut microbiome with high bacterial diversity and certain commensal pathogens (Faecalibacterium, *Akkermansia muciniphila*, and Ruminococcaceae family) correlates to T cell phenotypes that translate to favorable responses to ICI (98–101). Antibiotics can decrease microbiome diversity, and use of these agents prior to ICI decreases the likelihood of a favorable response to treatment in multiple cancers (102–104). The mechanism of action of the gut microbiome on responses to immunotherapy needs to be further elucidated.

Single-cell RNA sequencing revealed several subsets of T regulatory and cytotoxic, clonally expanded CD4 T cells in bladder cancer that were absent in patient-matched non-malignant tissue samples (105). The cytotoxic CD4 T cells were able to kill tumor cells in an MHC class II-dependent manner in cell culture (105). Additionally, a gene signature of cytotoxic CD4 T cells predicted response to PD-(L)1 blockade in a large cohort of metastatic bladder cancer patients (105).

### 5.2 Secondary resistance

#### 5.2.1 Gene mutations and loss of immunodominant neoantigens

Gene mutations leading to loss of immunodominant neoantigens or disruptions in immune effector signaling pathways are two major reasons for disease progression or relapse after initial response to ICI (**Figures 3G, H**). Comparison of pre-treatment and resistant tumors revealed that acquired resistance can be mediated by tumor cells that lose expression of immunodominant neoantigens or by an outgrowth of a subset of the tumor population that never expressed immunogenic neoantigens (106). Thus, epitope spreading (expanding the breadth of cancer antigens recognized by T cells) should combat secondary resistance by reducing immune pressure on a narrow set of antigens. Additionally, as summarized below, there are examples of several immune effector genes that have been rendered non-functional in patients who acquired resistance to PD-1 blockade. Specifically, in a study of four patients with melanoma whose disease initially responded to anti-PD-1 treatment but relapsed months to years later, two patients had homozygous loss-of-function mutations in their Janus kinase 1 (JAK1) or JAK2 genes (107). In the same study, a third patient had a truncating mutation in their β2-microglobulin gene (107). In another study, a patient who had metastatic uterine leiomyosarcoma experienced complete tumor regression in all but one lesion (108). Analysis of the germline tissue, responding tumors, and the resistant tumors revealed decreased gene expression of immunodominant neoantigens and homozygous loss of phosphatase and tensin homolog (PTEN) in the resistant tumor (108). This is significant because PTEN genetic deletion in a mouse model of melanoma led to increased levels of immunosuppressive cytokines and angiogenic factors (Vascular endothelial growth factor, VEGF), decreased T cell infiltration, decreased autophagy, and resistance to PD-(L)1 blockade (109). CRISPR-based screens could be a useful tool to identify immune evasion mechanisms in MCC.

#### 5.2.2 Lack of memory T cells

While this finding has been characterized in chronic viral infection and needs to be investigated in the context of cancer, it has been postulated that effector T cells that are responsible for initially controlling tumors do not differentiate into memory T cells (**Figure 3I**). Thus, the lack of cancer-specific memory T cells could mean that micro-metastases go unchecked and can give rise to new tumors. Indeed, MCC-specific T cells drop to very low or undetectable levels after effective disease control (21). This could be because cancer-specific T cells, similar to T cells that fight chronic infections, are antigen-addicted and cannot survive without disease burden and ongoing presentation of cognate peptide to TCRs. In a study of *Toxoplasma gondii* infection, a unique ‘transitional’ subset of T cells has been identified that possesses (1) hybrid effector and memory properties and (2) strong dependence on antigen for survival and proliferation to sustain the ongoing effector response (110). Additionally, while dysfunctional T cells can regain effector function *via* ICI, they are unable to differentiate into memory T cells (111, 112).

#### 5.2.3 Emerging mechanism: Cell-in-cell formations

A recent study demonstrated that cancer cells form transient cell-in-cell formations, where one tumor cell “hides” inside another tumor cell. This is in response to granules secreted by and cell surface proteins expressed on IFN-γ-activated CD8 T cells. The T cell cytokines and surface molecules induce upregulation of signal transducer and activator of transcription 3 (STAT3) and early growth response factor 1 (EGR1) transcriptional programs, which are necessary and sufficient for generating cell-in-cell formations. While the outer cell is killed by CD8 T cells, the inner cell is not susceptible to lytic granules secreted by cytotoxic T cells. Therefore, the inner cells can escape when the CD8 T cells are no longer in the microenvironment and give rise to new tumor cells, leading to resistance or partial responses to immunotherapy (113).

## 6 Discussion and future directions

Cancer-specific T cells are crucial for effective anti-tumor immunity and response to immunotherapy, but patient-specific mutations that drive most cancers make it difficult to develop reagents to identify and directly study the relevant cells. Given that virus-driven MCC contains compact, immunogenic oncoproteins and a low TMB, it offers a unique opportunity to relatively comprehensively identify cancer-specific T cells (**Figure 1**). Indeed, detailed functional studies have identified 20 immunogenic epitopes that can be presented by a variety of MHC alleles. Tetramers corresponding to the identified immunogenic epitopes have been used to identify cancer-specific T cells. These reagents have led to the discoveries that increased number, diversity, and functional avidity of cancer-specific T cells correlates to improved survival in MCC (**Figure 2**) **(** 36).

While immunotherapies that target T cell function have become a pillar of cancer treatment, only a subset of patients experience durable responses to PD-(L)1 pathway blockade (114). It is crucial to more fully understand the differences between patients who respond and those who do not to improve immunotherapies and prioritize patients for clinical trials of alternate and synergistic treatments. Many resistance mechanisms have been identified in MCC and other cancers, as discussed above (**Figure 3**). Specifically, MCPyV tetramer reagents have enabled studies that found that the frequency of circulating cancer-specific T cells before anti-PD-1 treatment predicts response (**Figure 2**) **(** 38, 39).

While PDX models have allowed pre-clinical testing of proposed treatments in immune-deficient mice, the fact that MCPyV oncoproteins are not capable of inducing tumors in mice has prevented basic cancer immunology studies. Importantly, more than a decade of effort has identified other conditions needed for MCC development in mice (induction of MCPyV oncoproteins, induction of neuroendocrine differentiation factor ATOH1, and deletion of one copy of tumor suppressor gene p53) (115). Using these findings, an immune competent mouse model of MCC has recently been developed (115). This mouse model can now synergize with a suite of improved bio-technological developments including better resolution for spatial transcriptomics, improved cancer-specific T cell identification methods *via* genetic and bioinformatic means, and increased integration of single-cell RNA sequencing with other cell state assays. As summarized below, these combined approaches should now allow us to: (1) further identify immune evasion mechanisms utilized by this immunogenic cancer; (2) characterize the cellular and signaling requirements of an effective anti-tumor response; and (3) determine the efficacy of novel and/or synergistic treatments.

### 6.1 Future direction: Identify additional immune evasion mechanisms

Many studies have suggested that infiltration of functional, cancer-specific T cells is linked to patient survival and response to immune-based treatments. Thus, one goal of identifying immune evasion mechanisms is to enhance the recruitment and activation of cytotoxic T cells that can kill tumor cells, effectively a turning a cold tumor (no immune cells) into a hot tumor (immune infiltrated). A recent study showed that pre-existing, clonally expanded, tissue resident memory T cells are early responders to neoadjuvant immunotherapy (75). However, the responding tumor-specific T cells are also present in patients who do not respond to immunotherapy (116). Spatial transcriptomic technologies are actively being improved to provide better resolution and obtain data at a single-cell level (117–119). Comparison of TMEs between responders and non-responders using these spatial technologies has the potential to reveal immune cells and secreted factors that are necessary for priming, activating, and supporting the cytotoxic function of cancer-specific T cells. Additionally, CRISPR-based genetic screens have identified a few immune evasion genes (summarized above in sections 5.1.3 and 5.2.1). Given the rapid rate of progress in the field, it may be possible in the near future to use CRISPR to both identify genes with loss-of-function mutations and then restore the function of these genes in a patient-specific manner, overcoming resistance to immunotherapy (120).

### 6.2 Future direction: Characterize the cellular and signaling requirements of an effective anti-tumor response

The rapid paced technological improvements in single-cell transcriptomics, especially the ability to generate multimodal data (e.g., RNA expression, cell surface protein expression, and assessing chromosome accessibility for the same cell) and associated analytical packages, have the potential to thoroughly characterize and compare cancer-specific immune cells across patients. Using these techniques, we can seek to (1) understand the phenotypic and TCR affinity differences between cancer-specific T cells that expand or contract over treatment; (2) uncover master regulator(s) of dysfunction and stem-ness for chronically activated T cells; and (3) better understand the influence of the microbiome on response to immunotherapy.

While peptide-MHC tetramers have been used for years to identify antigen-specific T cells, these reagents have several limitations: (1) they miss low-affinity TCRs that may be activated *in vivo* based on secondary signals that are not present *in vitro*; and (2) they may disassociate from the cell of interest prior to its detection in the assay, making it harder to distinguish signal from noise especially in single-cell sequencing applications. To counteract these limitations, new assays and bioinformatic tools that can rapidly identify cancer-specific T cells *via* expansion-based assays (22, 121), gene expression profiles (122, 123), and lentiviral fusion (27) are also being developed. However, these methods still require significant time and experimental expertise, in addition to large amounts of patient samples, and are thus unsuitable for clinical use. To extend these techniques for clinical use, it may be possible to draw from the fields of viral and bacterial immunology.

To identify T cells that are specific for CMV, SARS-CoV-2, and *Borrelia burgdorferi*, a bioinformatic “predictor” of TCR antigen specificity was developed for each of these pathogens (124–126). These classifiers were generated by comparing bulk TCRβ chain sequences from persons with or without the infection of interest. Statistical methods were used to identify T cells enriched in the patients harboring the infection, on the assumption that the expanded T cells recognize the pathogen of interest. However, without laboratory confirmation, statistical enrichment does not prove antigen specificity. By combining tetramer-, ENTER-, expansion-, or gene expression-based methods of identifying antigen-specific T cells, it should be feasible to develop predictors that can identify antigen-specific T cells from MCC patient samples in a clinically feasible manner.

### 6.3 Future direction: Determine the efficacy of novel and/or synergistic treatments

Understanding immune evasion and immunotherapy resistance mechanisms provides insight into potential alternate and synergistic treatments for patients unlikely to respond to individual agent immunotherapy. Indeed, several such clinical trials are already planned or under way, and advances in immunology techniques and bioinformatic tools will be crucial in determining the efficacy of these treatments for augmenting the number and/or function of cytotoxic CD8 T cells.

Given recent observations that the frequency of circulating cancer-specific T cells correlates with response to immunotherapy (38, 39), several potential therapeutic approaches can be considered: therapeutic vaccines, TCR-transgenic T cells, and *in vitro*-expanded cancer-specific T cells isolated from the blood or the tumor. Therapeutic vaccines are designed to induce an adaptive immune response against tumor antigens to effect tumor regression and eliminate minimal residual disease (127). Clinical trials targeting a variety of tumors are ongoing based on (1) sophisticated new approaches to select immunogenic tumor antigens *via* improved prediction algorithms and (2) advanced vaccine technologies (e.g., modified viral vector-based vaccines that co-express target antigens and immunostimulatory molecules) (128). Current therapeutic vaccine trials include one that targets virus-driven MCC (NCT05422781). This is a DNA vaccine that fuses an MCPyV oncoprotein with lysosomal-associated membrane protein-1 (LAMP1) to increase MHC class II antigen presentation and consequent priming of MCPyV-specific CD4 T cell responses (129). In addition to therapeutic vaccines, it may be possible to infuse *ex vivo* expanded or engineered tumor-specific lymphocytes. Indeed, T cells with a transgenic, high-affinity TCR for an MCPyV epitope are currently being tested in combination with standard-of-care immunotherapy and MHC class I upregulation (NCT03747484) (130). A recent study also tested the efficacy of *in vitro* expanded tumor-infiltrating lymphocytes (TIL) versus anti-CTLA-4 therapy in unresectable, late-stage melanoma (131). In this study, resected tumor samples were dissected, and TILs were expanded with anti-CD3 and IL-2. Patients who received TIL therapy experienced progression-free survival for 4 more months and overall survival for 7 more months compared to those treated with anti-CTLA-4 treatment (131). It remains to be seen whether TIL therapy has better efficacy than PD-1 pathway blockade.

In combination with checkpoint blockade, options that either increase the number of neoantigens to be targeted (via inhibition of DNA repair) or increase the number of cancer-specific T cells (via therapeutic vaccination) are being investigated. DNA damage response proteins have long been intriguing therapeutic targets as inhibiting their function could trigger cell death (132). Excitingly, preclinical studies of DNA damage response inhibitors also indicate that they induce cell death in an immunogenic manner and increase recruitment and activation of antigen-presenting cells (132–139). Recruitment of these antigen-presenting cells could prime adaptive immune responses to tumor antigens and, combined with ICI, lead to an enhanced anti-tumor cytotoxic response (132). Four different DNA damage response inhibitors (three of which target ataxia telangiectasia and Rad3-related, ATR) are currently being tested in combination with ICI in multiple cancers, and a clinical trial of an ATR inhibitor for MCC is upcoming (132). As discussed above, vaccine modalities have greatly improved, and several tumor vaccines are in preclinical and clinical stages of development (128). However, they often fail to provide clinical benefit due to immune evasion mechanisms employed by the tumor. Combination with immune checkpoint inhibitors could improve the ability of cancer-specific T cells elicited by the vaccine to exert anti-tumor effects (140).

It has also been observed that PD-1 pathway blockade often upregulates the expression of other immune checkpoint molecules (141, 142). Indeed, combination anti-CTLA-4 and anti-PD-1 treatment is currently offered to and beneficial for some patients who do not respond to anti-PD-1 monotherapy (143). However, anti-CTLA-4 treatment poses a higher risk of immune-related adverse events (144). Thus, alternate immune checkpoint targets have been identified and clinical trials testing the efficacy of combination treatments are underway (145). Specifically, the field is focusing on the immune checkpoint molecules LAG-3 and TIM-3. LAG-3 downregulates CD4+ T cell and myeloid cell responses *via* interaction with class II MHC on tumor cells and dendritic cells (146, 147). TIM-3 binding to galactin-9, a C-type lectin expressed on hematopoietic cells, leads to increased calcium influx-mediated cell death in TIM-3+ T cells (148). Thus, blocking these inhibitory cell surface proteins has the potential to rescue immune function in patients who do not respond to anti-PD-1 monotherapy. Indeed, numerous clinical trials are testing the efficacy of anti-TIM-3/anti-PD-1 or anti-LAG-3/anti-PD-1 therapies in a variety of solid tumors, with one of the anti-LAG-3 and anti-PD-1 combinations approved for first-line treatment of advanced melanoma in early 2022 (121, 149–151). Building on the success of dual checkpoint inhibition in other cancers, a clinical trial studying the efficacy of dual and/or triple ICI in MCC could also prove beneficial.

### 6.4 Concluding remarks

In contrast to most cancers driven by private, patient-specific mutations, Merkel cell carcinoma is driven by Merkel cell polyomavirus in most cases. Additionally, the MCPyV oncoproteins are highly immunogenic and thus tumors need to develop robust immune evasion mechanisms to ensure their own survival, often including dependence on PD-1 pathway activation. Indeed, anti-PD-1 immunotherapies are more effective in MCC than in any other solid cancer (152). The invariant, shared oncoproteins have allowed the development of tetramer reagents to identify cancer-specific T and B cells across patients. These reagents allow investigators to bypass the need for tumor antigen identification and to extract valuable information from small biological specimens. Thus, MCC is an excellent model for answering open questions in cancer immunology and immunotherapy. Specifically, recent improvements in single-cell RNA sequencing modalities, spatial transcriptomics, T cell identification methods, and the recently developed mouse model with an intact immune system should allow identification of additional immune evasion mechanisms and determination of efficacy of immunotherapies in pre-clinical studies. Virus-driven MCC provides a rich environment in which to study the intrinsic immune response to cancer as well as help understand why patients do and do not respond to current immunotherapy. The lessons learned from studying this tractable, immunogenic cancer may prove applicable to other cancers and yield significant benefits for cancer patients more broadly.

## Author contributions

CC and SJ performed literature search and manuscript preparation. CC, SJ and PN contributed to the article and performed editing. All authors approve the submitted version.

## Glossary

**Table:**

ADAR1	Adenosine Deaminase Acting on RNA 1
ATR	Ataxia telangiectasia and Rad3-related
C/EBP	CCAAT/enhancer binding protein
CLA	Cutaneous lymphocyte antigen
CTLA-4	Cytotoxic T lymphocyte-associated protein 4
EGR1	Early growth response factor 1
ER	Endoplasmic reticulum
HPV	Human papillomavirus
ICI	Immune checkpoint inhibition
IFN-γ	Interferon gamma
IL-2	Interleukin-2
IL-4	Interleukin-4
IL-10	Interleukin-10
JAK	Janus kinase
LAG-3	Lymphocyte activation gene 3
LPS	Lipopolysaccharide
MCC	Merkel cell carcinoma
MCPyV	Merkel cell polyomavirus
MHC	Major histocompatibility complex
MSI	Microsatellite instability
NK	Natural killer
PD-1	Programmed cell death protein 1
PD-L1	Programmed death ligand 1
PTEN	Phosphatase and tensin homolog
PTPN2	Protein tyrosine phosphatase non-receptor 2
PDX	Patient-derived xenograft
SETDB1	SET Domain Bifurcated Histone Lysine Methyltransferase 1
STAT3	Signal transducer and activator of transcription 3
STING	Stimulator of interferon genes
TAP	Transporter associated with antigen presentation
TBK1	Tank-binding kinase 1
TCR	T cell receptor
TGF-β1	Transforming growth factor beta 1
TIGIT	T cell immunoreceptor with Ig and ITIM domains
TIL	Tumor-infiltrating lymphocytes
TIM-3	T cell immunoglobulin and mucin domain 3
TLR9	Toll-like receptor 9
TLS	Tertiary lymphoid structures
TME	Tumor microenvironment
TMB	Tumor mutational burden
UV	Ultraviolet
VEGF	Vascular endothelial growth factor
WES	Whole exome sequencing
